# Ultrasound-microbubble targeted delivery of ICAM-1 in mouse model of peripheral arterial disease

**DOI:** 10.1186/2050-5736-3-S1-P56

**Published:** 2015-06-30

**Authors:** Lena Badr

**Affiliations:** 1 University of Virginia, Charlottesville, Virginia, United States

## Background/introduction

Peripheral arterial disease (PAD) affects 12 million people in the United States. Unfortunately, current treatments are highly invasive and have limited efficacy. Ultrasound (US) in conjunction with microbubbles (MB) has recently been explored as a non-invasive strategy for the delivery of intravascularly circulating therapeutic agents.

US mediated MB oscillation can lead to non-damaging, reversible and localized vascular permeabilization resulting in substantial increases of nanoparticle (NP) concentrations in US-treated tissue. Furthermore, by coating NPs with a brush layer of polyethylene glycol (PEG), NPs have long circulating half-lives and limited toxicity. This study investigates the ability of US to deliver ICAM-1-bearing PEG/polyethylenimine (PEG/PEI) NPs to the vascular endothelium in a mouse model of PAD. ICAM-1, an adhesion molecule, expressed post-occlusion by the endothelium of collateral arteries has previously been shown to aid in blood flow restoration. Overexpression of ICAM-1 in this model aims to re-establish pre-occlusion blood flow non-invasively.

## Methods

C57BL/6 mice were secured supine and their hind limbs were depilated. A 0.75’’ diameter 1 MHz unfocused US transducer was ultrasonically coupled. The tail vein was cannulated and a coinjection of MBs and 40 μg of ICAM-1 and luciferase bearing NPs was coincident with US treatment. MBs were injected at a dosage of 105/g. Luciferase and ICAM-1 transgenes were under control of the beta-actin promoter. Sonications were performed with a 0.015% duty cycle, a sonication time of 12 minutes, and a peak negative pressure of 0.6 MPa. Three days post US treatment, mice underwent femoral artery ligation (FAL) surgery to simulate an occlusion. Luciferase expression was assessed through bioluminescent imaging following a 150 mg/kg injection of luciferin in an *In Vivo* Imaging System at 3, 5, 7, 10, and 14 days after US treatment. Following euthanasia, the animal was perfused transcardially with paraformaldehyde and microfil and whole-mounted or sectioned. Sections were immunostained for smooth muscle alpha actin and ICAM-1 to further investigate ICAM-1 expression in collateral arterial endothelial cells.

## Results and conclusions

Delivery of luciferase-bearing PEG/PEI NPs resulted in robust, localized bioluminescence through day 14, the last day tested. Bioluminescence was not detected on the contralateral non-treated limb. Bioluminescence peaked 7 days after US treatment. US mediated vascular permeabilization presents a novel platform for drug and gene delivery technologies. Our results indicate that robust, localized transgene expression can be achieved in a mouse model of PAD. Currently, we are investigating the potential for enhanced US-mediated ICAM-1 gene therapy.

**Figure 1:**
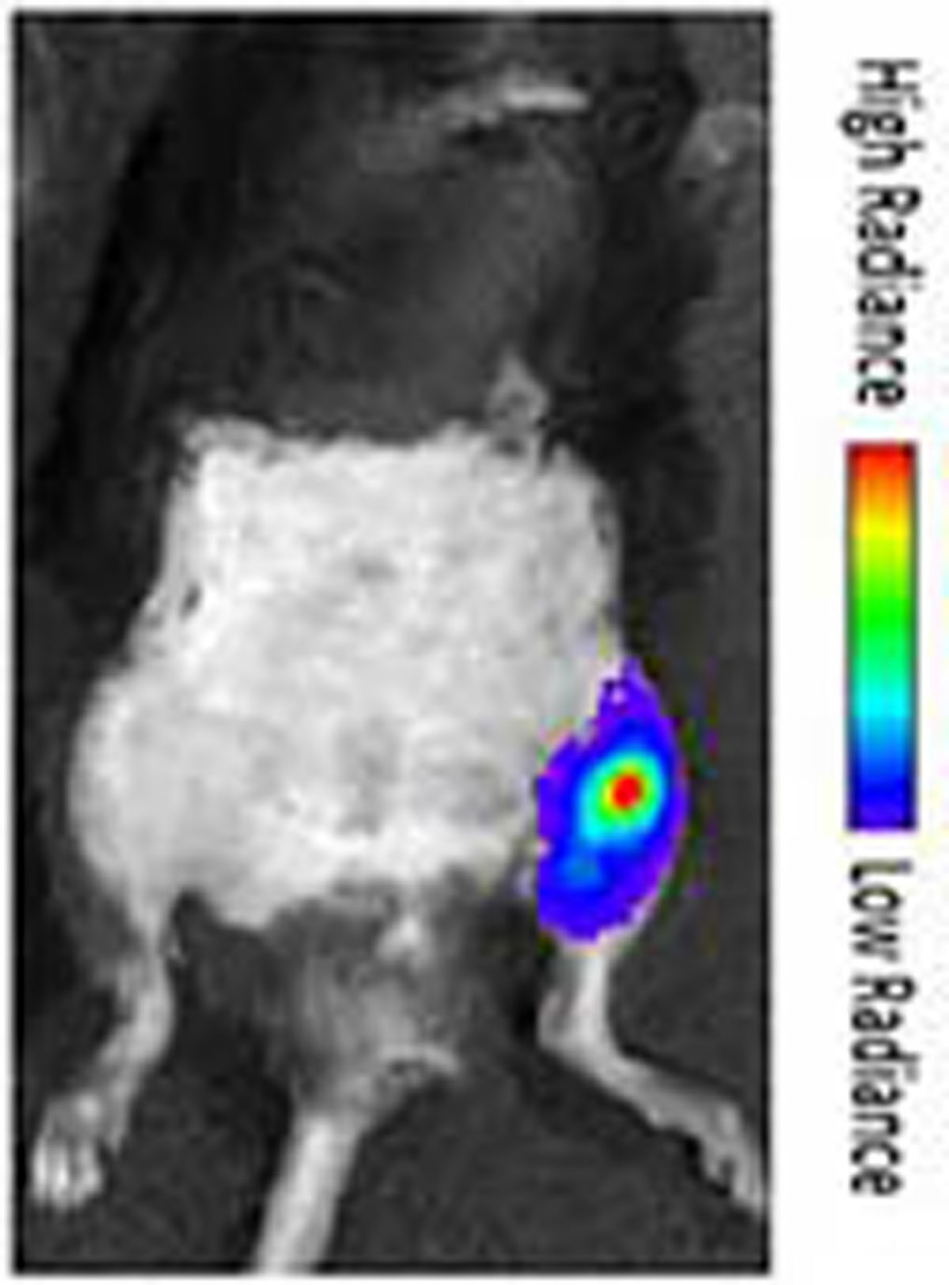
Localized bioluminescence shown in sonicated left hind limb. Bioluminescence was measured on day 7 after US treatment. No bioluminescence was detected on contralateral non-sonicated limb.

